# Obsessive-compulsive and posttraumatic stress symptoms among civilian survivors of war

**DOI:** 10.1186/s12888-016-0822-9

**Published:** 2016-04-27

**Authors:** Naser Morina, Vita Sulaj, Ulrich Schnyder, Richard Klaghofer, Julia Müller, Chantal Martin-Sölch, Michael Rufer

**Affiliations:** Department of Psychiatry and Psychotherapy, University Hospital Zurich, University of Zurich, Culmannstrasse 8, 8091 Zürich, Switzerland; Psychiatric Hospital Königsfelden, Brugg, Switzerland; Psychiatric Services Thurgau, Münsterlingen, Switzerland; Division of Clinical and Health Psychology, Department of Psychology, University of Fribourg, Fribourg, Switzerland

**Keywords:** Obsessive-compulsive symptoms, Trauma, Post-traumatic stress, Civilian war survivors, Refugee

## Abstract

**Background:**

Several psychological sequelae have been identified in civilian war survivors. However, little is known about the prevalence of obsessive-compulsive symptoms and their relationship to trauma in this population.

**Method:**

Fifty-one adult civilian survivors of the Kosovo War (28 males) who had immigrated to Switzerland completed the Revised Obsessive-Compulsive Inventory Scale, the Posttraumatic Stress Diagnostic Scale and the Hopkins Symptom Checklist. Data were analysed using multiple regression analyses.

**Results:**

Overall, 35 and 39 % of the sample scored above the cut-offs for likely obsessive-compulsive disorder and posttraumatic stress disorder, respectively. Participants with high levels of posttraumatic stress symptoms were significantly more likely to have obsessive-compulsive symptoms, and vice versa. In multiple regression analysis, gender and severity of posttraumatic stress symptoms were predictors of obsessive-compulsive symptoms, whereas number of traumatic life event types and depressive symptoms were not.

**Conclusion:**

Given the small sample size, the results of this study need to be interpreted cautiously. Nevertheless, a surprisingly high number of participants in our study suffered from both obsessive-compulsive and posttraumatic stress symptoms, with obsessive-compulsive symptoms tending to be more pronounced in women. It remains, therefore, critical to specifically assess both obsessive-compulsive and posttraumatic stress symptoms in civilian war survivors, and to provide persons afflicted with appropriate mental health care.

## Background

In recent years, increasing attention has been given to the long-term psychological consequences of war-related trauma in civilian populations, e.g. survivors of the Balkan wars [10, 29, 35, 43]. Studies performed in Bosnia [1, 22], Croatia [44, 45], Serbia [39, 44] and Kosovo [11, 36, 39, 48] indicate current rates of 57 % for depression and 13–36 % for posttraumatic stress disorder (PTSD) in the civilian population. Moreover, up to 75 % of people with current PTSD have been reported to have at least one comorbid psychiatric disorder [1].

Beyond that, the relationship between war-related experiences and the occurrence of other mental health disorders such as obsessive-compulsive disorder (OCD) in civilians has begun to receive attention. Studies and case reports of civilians and veterans indicate that elevated posttraumatic stress and obsessive-compulsive symptom levels frequently co-occur in individuals with a history of traumatic exposure [26] and that trauma may play a major role in the development of OCD [7]. The prevalence of OCD has been reported to be up to 47 % in people with PTSD, compared to approximately 1 % in the general population [23, 26, 38]. On the other hand, up to 54 % of individuals with OCD reported having experienced at least one traumatic life event [6, 17] and indicated higher levels of posttraumatic stress symptoms [15, 25, 50]. In contrast, Grabe et al. [21] compared 210 OCD patients with 133 gender- and age-matched controls in a German multi-centre study, and they identified a higher lifetime rate of past severe traumatization in controls than in OCD patients. The investigators also reported low rates of trauma-related disorders either before or within the same year as OCD onset, at 2.9 and 1.5 %, respectively.

OCD and PTSD share several common elements of clinical symptomatology, with the occurrence of intrusive images or thoughts being common in both conditions. Whereas OCD patients tend to experience intrusive images of distressing events that may befall themselves or others, PTSD patients typically experience recurrent memories of their traumatic experience. Both disorders tend to result in responses to the distress, including avoidance behaviours or rituals [2, 47]. Various authors have described a “posttraumatic obsessive-compulsive disorder” [15, 16, 42, 47] in which the onset of OCD is clearly associated with the experience of a potentially traumatic event. Furthermore, studies indicate that patients with comorbid OCD and PTSD may be less responsive to psychotherapy than OCD patients without PTSD [18, 19].

Based on these studies, it could be hypothesized that war-related posttraumatic OCD can lead to high levels of mental health problems among civilian survivors of war. Therefore, there is a need to understand the relationship between both disorders, particularly as targeted therapy could lead to a lessening and eventually a decrease of the obsessive-compulsive symptoms [40]. A large representative study performed in five Balkan countries five to 15 years after the war in the Balkans estimated the prevalence of OCD among civilian post war survivors as between 0.2 and 6.1 % [43]. In a study of bereaved and non-bereaved war survivors in Kosovo, Morina et al. [37] identified OCD rates of 3.4 and 0.6 %, respectively. However, to our knowledge, there are no published studies that have specifically assessed obsessive-compulsive symptoms among survivors of war after migration to a new host country. Furthermore, research suggests that mental health problems in refugees and migrants persist for many years after their first occurrence [49] and that comorbidity presents a therapeutic challenge. Therefore, the main objectives of this study were to assess the severity and characteristics of obsessive-compulsive symptoms among survivors of the 1998–1999 Kosovo War living in Switzerland and to identify possible associations between obsessive-compulsive and posttraumatic stress symptoms. Finally, as PTSD has been reported to be strongly associated with the severity of depressive symptoms, we will additionally test whether PTSD or depressive symptoms alone or in interaction with each other account for OCD symptoms.

## Methods

### Participants recruitment

Data were collected between October 2010 and October 2011. To be eligible for participation, individuals had to have emigrated from Kosovo to Switzerland during or after the Kosovo War, which ended in 1999. All participants were adults who spoke fluent Albanian and were either (1) respondents to an advertisement published in an Albanian-language newspaper distributed in Switzerland; (2) parents of students at an Albanian-speaking school for Kosovar immigrants; (3) respondents to a website for Kosovar immigrants; or (4) patients of an Albanian-speaking physician. The study was announced as follows: *“War can lead to mental and physical health problems. To investigate the war-related consequences of the Kosovo War we are conducting a study at our outpatient clinic. If you have immigrated during or after the War, please e-mail or call us. A study member will contact you for further details.”*

Exclusion criteria were as follows: age below than 18 years old; lack of war-time experience; immigration before the Kosovo War; and possession of significant military rank (i.e., non-civilian). All participants provided written informed consent prior to study participation in accord with the Declaration of Helsinki. The study protocol was approved by the ethics committee of the canton of Zurich, Switzerland.

### Data collection

Data were collected within the context of a cross-sectional survey. All interviews were conducted at the participants’ home by a clinical psychologist fluent in both Albanian and German, and interviews were conducted in either language, according to the preference of the participant. The instruments were translated into Albanian by a bilingual mental health professional and then back-translated into the source language following gold-standard translation practices [3]. Both copies were sent to a native Albanian-speaking psychiatrist who compared the translations and recommended revisions where necessary. The feasibility and comprehensibility of the measures were checked in a pilot phase and found to be acceptable. Interview time generally ranged from 60 to 90 min, and participants were reimbursed CHF 30 (approximately US$ 30) for participation. Demographic data, information on each participant’s war-time experiences, and date of immigration to Switzerland were collected to verify eligibility.

Fifty-one participants were included in the study. Of these, 28 (54.9 %) were male, and the mean age was 43.0 (SD = 5.4) years. The average time since emigration from Kosovo to Switzerland was 11.3 (SD = 1.4) years. Nineteen of 51 (37.3 %) were working full-time at the time of assessment, and an equal number were unemployed. The remaining thirteen (25.5 %) were working part-time. Further demographic information is presented in Table 1.

**Table 1:** Description of the sample of Kosovo emigrants (*N* = 51)

Variable	N (%)	M (SD)	Range
Gender - male	28 (54.9 %)		
Age (years)		43.0 (5.4)	32–54
Working (full-time or part-time)	32 (62.7 %)		
Education (years)		9.29 (4.46)	0–20
Time since immigration (years)		11.3 (1.4)	(6–13)
OCD overall severity		32.63 (13.14)	18–90
Washing		6.24 (2.78)	3–15
Obsessions		6.12 (3.96)	3–15
Hoarding		5.37 (3.09)	3–15
Ordering		4.88 (2.62)	3–15
Checking		5.25 (2.60)	3–15
Mental neutralizing		4.76 (2.39)	3–15
Number of TLE		10.3 (4.9)	2–28
PTSD severity		12.4 (13.1)	0–51
Depression		3.7 (1.9)	1–4
Diagnoses			
Probable diagnosis of OCD	18 (35.3)		
Probable diagnosis of PTSD	20 (39.2)		
Clinically relevant depression	23 (45.1)		

*Notes*: *TLE* traumatic life event types (experienced and/or witnessed; HTQ and PDS), *OCD* obsessive-compulsive disorder (OCI-R), *PTSD* posttraumatic stress disorder (PDS); depression: HSCL-25

### Obsessive-compulsive symptoms

Obsessive-Compulsive Symptoms were assessed using the revised version of the Obsessive-Compulsive Inventory (OCI-R) scale [14]. The 18-item questionnaire asked three questions in each of six OCD domains: washing, checking, obsessions, mental neutralizing, ordering, and hoarding. For each item, individuals were asked to rate their degree of distress with a particular behaviour, using the following five response options: 1 = *not at all*; 2 = *a little*; 3 = *moderately*; 4 = *a lot*; 5 = *extremely*. For each of the six domains, scores ranged from a minimum of 3 points to 15 points. A summation score of the six domains was also calculated (range: 18–90). A total score of 42 or more indicated the presence of clinically significant OCD symptoms [14]. This scale has been used in culturally diverse contexts [20, 54]. Cronbach’s alpha for the present study was .92.

### Potentially traumatic events and posttraumatic stress symptoms

The *Posttraumatic Diagnostic Scale (PDS)* [13] is a 49-item self-report questionnaire, designed for adults, that measures the presence and severity of PTSD on the basis of the DSM-IV criteria. Part I is a trauma checklist that inquired about several types of traumatic events. For the purposes of this study, the PDS checklist was modified by adding traumatic event types from the Harvard Trauma Questionnaire that frequently occur in refugees [32], resulting in a total of 23 items measuring traumatic life event types (i.e. brainwashing, torture, being close to death), each rated as either experienced, witnessed, both, or neither. Part III assessed 17 potential PTSD symptoms experienced in the month prior to assessment not related to a specific event. Respondents were asked to rate the severity of each symptom from 0 (*not at all/only once*) to 3 (*5 or more times a week/almost always*). The PDS yielded a total severity score (ranging from 0 to 51) that largely reflected the frequency of the 17 symptoms of PTSD. From this summation score, a categorical diagnosis of PTSD was made using a pre-set, published algorithm that follows the DSM-IV diagnostic criteria for PTSD [12]. This scale evidenced excellent internal consistency (α = .98).

### Depression

The second section of the Hopkins Symptom Checklist, HSCL-25, [34] consisting of 15 items, was used to measure depression symptoms. The HSCL-25 has been translated into many languages and used in different cultural contexts [33, 52]. The items are rated on a 4-point Likert scale summing up to a score for the average, ranging from 1 to 4 [34]. The cut-off score is set at 1.75 points and was evaluated in relation to different diagnostic psychiatric interviews. It has been found to be a reliable measure of the presence of a depressive disorder [33]. Internal consistency for the scale in the present study was .97.

### Data analysis

Rates of OCD, PTSD and depression were calculated according to the cut-offs for each instrument (OCI-R, PDS and HSCL). Pearson’s correlation coefficients were calculated to identify associations between individual symptoms. Multiple linear regression (using the enter method) was utilized to examine the relationship between obsessive-compulsive symptoms as the dependent variable and gender, traumatic life event types (TLE), PTSD severity and depression as independent variables. Assumptions for regression analyses were checked in terms of linearity of the relationships between OCD and independent variables, autocorrelation of residuals (Durbin-Watson-test = 2.430), and homoscedasticity and normal distribution of residuals, and all were found to be satisfactory. Furthermore, we found no multicollinearity between independent variables (all VIF < 9.7). The results are reported in terms of *b* (SE(*b*)), Beta (95 % CI) and adjusted *R*-squared. To examine whether gender was a moderator in the relationship between OCD and symptoms of PTSD, the interaction between gender and PTSD severity was included in the model. A moderator analysis showed no significant interaction (*p = .568*), i.e., gender was not a significant moderator. All analyses were conducted using IBM SPSS Statistics Version 21 software (SPSS Inc., Chicago, IL, USA).

## Results

### Lifetime rates of traumatic events and elevated levels of psychopathological symptoms

Participants reported having experienced and/or witnessed a mean of 10.3 different types of potentially traumatic events (PTE) during their lifetimes (SD = 4.9). Most frequently reported included “lack of shelter” (98 %), “combat situations” (96.1 %), “lack of food and water” (94.1 %), and “ill health without access to medical care” (58.8 %). The least frequently reported PTEs included sexual violence (Table 2). Both obsessive-compulsive and posttraumatic stress symptoms were common, with 18 of 51 (35 %) scoring above the cut-off for OCD and 20 of 51 (39 %) scoring above the cut-off for PTSD. Among the six core OCD symptoms in the overall sample, compulsive washing was the most common and the most severe (M = 6.24, SD = 2.80), followed by obsessions (M = 6.12, SD = 3.96). Men and women differed in the rank order of their symptoms, their overall OCD score (*p =* .024), and their OCD sub-scores, with women rating their obsessive-compulsive distress higher. Overall, almost half of 23 women (47 %) scored above the cut-off for OCD versus one in four men (7 out of 28, 25 %; *p =* .08). Men and women did not significantly differ in terms of the number of experienced traumatic life event types (*p =* 0.58), their overall scores, or the percentage scoring above the cut-off for PTSD (*p =* .603) or depression symptoms (*p =* .138). A significant negative correlation was found between education and all mental health symptoms, and occupation and mental health symptoms, respectively (Table 3).

**Table 2:** Potentially traumatic events as experienced or witnessed (lifetime; *N* = 51)

	N (%)
Lack of shelter	50 (98.0)
Combat situation	49 (96.12)
Lack of food or water	48 (94.1)
Ill health without access to medical care	30 (58.8)
Forced separation from family member	26 (51.0)
Murder of a family member or friend	21 (41.2)
Unnatural death of a family member or friend	19 (37.3)
Murder of one or more strangers	16 (31.4)
Serious physical injury	15 (29.4)
Imprisonment	13 (25.5)
Enforced isolation from others	12 (23.5)
Being close to death	11 (21.6)
Life-threatening illness	8 (15.7)
Torture	7 (13.7)
Natural disaster	6 (11.8)
Serious accident, fire or explosion	5 (9.8)
Brainwashing	2 (3.9)
Non-sexual assault by a stranger	1 (2.0)
Non-sexual assault by family member or someone known	1 (2.0)
Sexual contact when you were younger than 18 with someone who was 5 or more years older than you	1 (2.0)
Sexual assault by a family member or someone you know	0 (0.0)
Sexual assault by a stranger	0 (0.0)
Disappearance or kidnapping	0 (0.0)

**Table 3:** Correlations between observed variables (*N* = 51)

	Demographics					Obsessive-compulsive symptoms							Trauma and post-traumatic stress symptoms				Depression
	1	2	3	4	5	6	7	8	9	10	11	12	13	14	15	16	17
1 Age	-																
2 Sex	-.190	-															
3 Education	-.034	-.542^**^	-														
4 Occupation	.053	-.343^*^	.630^**^	-													
5 Stay in CH	.131	-.078	.225	.210	-												
6 OCD Washing	-.049	.266	-.453^**^	-.670^**^	-.116	-											
7 OCD Checking	.001	.216	-.424^**^	-.604^**^	-.025	.669^**^	-										
8 OCD Obsessions	.100	.083	-.520^**^	-.666^**^	.026	.767^**^	.726^**^	-									
9 OCD Mental neutralizing	-.035	.174	-.294^*^	-.388^**^	-.047	.382^**^	.455^**^	.449^**^	-								
10 OCD Ordering	-.046	.345^*^	-.394^**^	-.635^**^	-.067	.476^**^	.539^**^	.435^**^	.577^**^	-							
11 OCD Hoarding	.023	.393^**^	-.464^**^	-.435^**^	-.266	.488^**^	.185	.245	.246	.513^**^	-						
12 OCD total score	.010	.317^*^	-.577^**^	-.761^**^	-.106	.854^**^	.792^**^	.833^**^	.660^**^	.763^**^	.595^**^	-					
13 TLE	.036	-.267	-.027	-.038	.187	.202	.086	.219	.126	-.023	-.116	.117	-				
14 PTSD Re-experiencing	.097	.062	-.513^**^	-.683^**^	-.126	.713^**^	.631^**^	.871^**^	.513^**^	.470^**^	.292^*^	.793^**^	.380^**^	-			
15 PTSD Avoidance	.102	.070	-.555^**^	-.649^**^	-.152	.747^**^	.656^**^	.870^**^	.495^**^	.443^**^	.289^*^	.796^**^	.352^*^	.935^**^	-		
16 PTSD Hyperarousal	.072	.070	-.514^**^	-.684^**^	-.190	.747^**^	.745^**^	.885^**^	.523^**^	.513^**^	.306^*^	.845^**^	.202	.923^**^	.918^**^	-	
17 PTSD severity	.098	.075	-.543^**^	-.691^**^	-.151	.758^**^	.698^**^	.898^**^	.525^**^	.490^**^	.307^*^	.834^**^	.322^*^	.975^**^	.977^**^	.973^**^	-
18 Depression	.104	.211	-.599^**^	-.690^**^	-.143	.742^**^	.732^**^	.876^**^	.587^**^	.518^**^	.283^*^	.842^**^	.195	.880^**^	.920^**^	.899^**^	.924^**^

*Note*: *p < 0.05; **p < 0.01, *p*-values are unadjusted. CH: Switzerland (mean years since arrival); *TLE* traumatic life event types (experienced and witnessed), *OCD* obsessive-compulsive disorder (OCI-R), *PTSD* posttraumatic stress disorder (PDS); depression: HSCL-25

### Associations between obsessive-compulsive and posttraumatic stress symptoms

A strong correlation (*r* = .834; *p* < .001) was found between obsessive-compulsive and posttraumatic stress symptoms. Sixteen of 20 participants with elevated levels of PTSD symptoms (80 %) also met cut-off criteria for likely OCD, and 16 of 18 with elevated levels of OCD symptoms (89 %) also met cut-off criteria for PTSD. This significant association persisted when correlation analysis was performed on the different OCD sub-scores and the PTSD core symptoms, with moderate to strong positive correlations, as shown in Table 3. With regard to the relationship between depressive and obsessive-compulsive symptoms, similar associations (OCD in depression = 94 %; depression in OCD = 74 %; *r* = .842; *p* < .001) were found. However, no significant correlations were identified between the number of traumatic life event types and any OCD symptoms. Multiple regression analysis (Table 4) revealed that obsessive-compulsive symptoms were mainly explained by gender and PTSD severity, but not by traumatic life event types and depression. The final model accounted for 76 % of the variance in obsessive-compulsive symptoms.

**Table 4:** Summary of multiple regression analysis for participants’ OCD scores (*N* = 51)

Variable	*B*	*(SE B)*	*β*	*(95 % CI)*	*t*
Sex	5.027	(2.439)	.192*	(0.01; 0.38)	2.061
Education	-.013	(.314)	-.004	(−0.21; 0.20)	-.041
Occupation	−1.862	(1.356)	-.112	(−0.27; 0.05)	−1.373
TLE	-.112	(.222)	-.042	(−0.21; 0.12)	-.507
PTSD severity	.534	(.218)	.533*	(0.11; 0.96)	2.446
Depression	4.204	(3.112)	.275	(−0.12; 0.68)	1.351
*Adjusted R* ^*2*^	.755				
*F (6,44)*	26.721				
*p*	<.001				

*Note*: *p < 0.05; *TLE* traumatic life event types (experienced and/or witnessed)

## Discussion and Conclusions

In the present study, we examined obsessive-compulsive symptoms and their associations with posttraumatic stress and depression in civilian survivors of war living outside their country of origin. To our knowledge, this is the first study that assessed the relationship of obsessive-compulsive symptoms and traumatic experiences in civilian war survivors a decade after the war had ended. High levels of obsessive-compulsive symptoms were found, affecting roughly one in three in our sample, with significantly higher levels in women. Our main finding was a strong association between OCD and PTSD symptoms, with more than 80 % of the individuals reporting symptoms of one disorder also displaying symptoms of the other. In multiple regression analysis, obsessive-compulsive symptoms were mainly related to gender and PTSD severity, but not to potentially traumatic events and depression.

Our results confirm several previously published studies that have identified significant associations between OCD and both traumatic exposure and PTSD [25, 26], as well as between obsessive-compulsive symptoms and childhood trauma [27, 30]. Our results are similar to Grabe et al.’s [21] study, which did not find any significant associations between trauma and OCD. However, in contrast to the Grabe et al. study, our sample was characterized by a high number of self-reported potentially traumatic events. This might be one explanation for the different findings. Furthermore, our population was recruited from a community sample, as opposed to a psychiatric or a clinical sample.

Several explanations could account for the frequent co-occurrence of obsessive-compulsive and posttraumatic stress symptoms (see review; [8]). Cognitive models of PTSD posit that negative appraisals lead to the exaggerated interpretation of stimuli as threatening, which results in elevated distress [9]. Following war, affected civilians may develop cognitive patterns involving concerns about safety, contamination, or the need to hoard, which may in turn lead to compulsive rituals to reduce arousal [46]. In the wake of war in particular, it is possible that threats to one’s safety may trigger specific OCD-related concerns. For example, people may develop excessive checking rituals to maintain their sense of safety. Alternatively, those exposed to sexual violence or body dismemberment may develop concerns regarding contamination. Further, Gershuny and colleagues [19] postulate that OCD and PTSD are two disorders on the same continuum. Thus, the authors suggest that there is a substantial overlap between the symptomatologies of OCD and PTSD, meaning that both disorders are characterized by anxiety-provoking recurrent and intrusive thoughts. There is also evidence of the link between trauma experience and OCD being moderated by genetic factors that are similarly implicated in PTSD development, raising the possibility of shared genetic risk factors for developing both conditions following trauma [24].

Of note, women reported more obsessive-compulsive symptoms. Men and women have the disorder with equal frequency in epidemiological studies [53]. Despite this apparent similarity, there is evidence that gender plays a relevant role in OCD phenotypic expression [31]. Considering that we studied obsessive-compulsive symptoms in a sample who fled significant trauma experienced during years of war, it is possible that exposure or response to traumatic events occurred differentially across genders in this sample. Further, we note that admission of OCD symptoms is sometimes hindered by embarrassment about the nature of the images or impulses experienced, and this may have particularly impacted responses from female participants.

The high percentage of individuals scoring above the cut-off for probable PTSD (39 %) and clinically relevant depression (45 %) should also be noted. This finding is remarkable because this was not a treatment-seeking sample, and participants were interviewed more than ten years after the war while living in a safe context. We note, however, that there is good evidence that the rate of psychiatric disorders in refugee groups tends to be elevated in small samples [51]. Accordingly, it is possible that a study with a larger and more representative sample of Kosovar war survivors living in Switzerland would yield lower psychopathology rates. Having said that, the literature suggests that after exposure to a traumatic event, the co-occurrence of PTSD and other disorders is almost the rule and not an exception [4]. The high correlation between post-traumatic stress, obsessive-compulsive symptoms and clinically relevant depression in the current sample can be understood under this perspective.

However, we found no gender differences regarding exposure to potentially traumatic events and probable PTSD in the current sample, which is in contrast to the epidemiological literature, but has been shown in samples of military personnel [41]. This somewhat surprising finding may be explained by comparable trauma exposure in men and women during and after the war.

A further result to be noted is the high negative correlation between occupation and mental health symptoms and education and mental health symptoms, respectively. Those two factors seem to be as protective factors and are in line with literature for obsessive-compulsive symptoms [5], posttraumatic stress symptoms [55] and depression [28].

Several limitations suggest that these preliminary results should be interpreted with caution. First, given the small sample size we cannot make any firm statements with regard to the representativity of our sample. Also, we cannot rule out the possibility that respondents to the various recruitment efforts had a vested interest in the study. In other words, the high levels of likely OCD, probable PTSD and clinically relevant depression might have been influenced by the sampling process. Second, our sample was restricted to those who had left Kosovo to immigrate to Switzerland, a group that may be psychologically and demographically different from those who chose to remain in Kosovo; a study of comparable data in a parallelized sample of participants still living in Kosovo would be of interest. Third, given the cross-sectional design of our study, we cannot make any inferences regarding the sequence of OCD, PTSD, or depression symptom onset in relation to each other. Relatedly, this study did not assess the extent to which the symptoms existed before the war. Fourth, while we used measures that have been validated across cultural groups, they have not been validated in Albanian-speaking samples. Further, we did not conduct clinical interviews, and consequently the extent to which self-reported symptom levels of OCD, PTSD and depression would correspond to clinical diagnoses is unclear. Finally, the fact that symptoms of OCD, PTSD and depression correlated highly in the current sample might suggest that participants were suffering from an underlying general distress that led to an over-endorsement of all psychiatric symptoms.

Taken together, our results suggest that the screening of war-related traumatized populations should not be restricted to and focused on PTSD symptoms, but should also take into account other mental disorders or psychopathological symptoms, such as obsessive-compulsive and depressive symptoms. As symptomatology of PTSD-related intrusions is in some ways similar to obsessive images and thoughts and depressive ruminations, differential diagnosis of OCD, PTSD and depression should be considered. Further investigations with respect to the role of traumatic experiences, such as war, in OCD aetiology are needed because post-traumatic OCD may be seen as a specific subtype, which has clinical implications. In consequence it would be important to provide specific support and treatment aimed at reducing long-term posttraumatic psychosocial disability, including obsessive-compulsive symptoms.

### Ethics approval and consent to participate

The study protocol was approved by the ethics committee of the canton of Zurich, Switzerland (Project Nr. E-18/2009). All participants provided written informed consent prior to study participation.

### Consent for publication

Not applicable.

### Availability of data and materials

Data will be shared upon request.

## References

[1] Avdibegovic E, Hasanovic M, Selimbasic Z, Pajevic I, Sinanovic O (2008). Mental health care of psychotraumatized persons in post-war Bosnia and Herzegovina--experiences from Tuzla Canton. Psychiatr Danub.

[2] Badour CL, Bown S, Adams TG, Bunaciu L, Feldner MT (2012). Specificity of fear and disgust experienced during traumatic interpersonal victimization in predicting posttraumatic stress and contamination-based obsessive-compulsive symptoms. J Anxiety Disord.

[3] Bontempo R (1993). Translation fidelity of psychological scales: an item response theory analysis of an individualism-collectivism scale. J Cross-Cult Psychol.

[4] Brady KT, Killeen TK, Brewerton T, Lucerini S (2000). Comorbidity of psychiatric disorders and posttraumatic stress disorder. J Clin Psychiatry.

[5] Cath DC, van Grootheest DS, Willemsen G, van Oppen P, Boomsma DI (2008). Environmental factors in obsessive-compulsive behavior: evidence from discordant and concordant monozygotic twins. Behav Genet.

[6] Cromer KR, Schmidt NB, Murphy DL (2007). An investigation of traumatic life events and obsessive-compulsive disorder. Behav Res Ther.

[7] de Silva P, Marks M (1999). The role of traumatic experiences in the genesis of obsessive–compulsive disorder. Behav Res Ther.

[8] Dykshoorn KL (2014). Trauma-related obsessive-compulsive disorder: a review. Health Psychol Behav Med.

[9] Ehlers A, Clark DM (2000). A cognitive model of posttraumatic stress disorder. Behav Res Ther.

[10] Eytan A, Bischoff A, Rrustemi I, Durieux S, Loutan L, Gilbert M, Bovier PA (2002). Screening of mental disorders in asylum-seekers from Kosovo. Aust N Z J Psychiatry.

[11] Eytan A, Guthmiller A, Durieux S, Loutan L, Gex-Fabry M (2011). Mental and physical health of Kosovar Albanians in their place of origin: a post-war 6-year follow-up study. Soc Psychiatry Psychiatr Epidemiol.

[12] Foa EB (1995). PDS (posttraumatic stress diagnostic scale) manual.

[13] Foa EB, Cashman L, Jaycox L, Perry KJ (1997). The validation of a self-report measure of posttraumatic stress disorder: the posttraumatic diagnostic scale. Psychol Assess.

[14] Foa EB, Huppert JD, Leiberg S, Langner R, Kichic R, Hajcak G, Salkovskis PM (2002). The obsessive-compulsive inventory: development and validation of a short version. Psychol Assess.

[15] Fontenelle LF, Cocchi L, Harrison BJ, Shavitt RG, do Rosario MC, Ferrao YA, Torres AR (2012). Towards a post-traumatic subtype of obsessive-compulsive disorder. J Anxiety Disord.

[16] Fostick L, Nacasch N, Zohar J (2012). Acute obsessive compulsive disorder (OCD) in veterans with posttraumatic stress disorder (PTSD). World J Biol Psychiatry.

[17] Fricke S, Köhler S, Moritz S, Schäfer I (2007). Early traumatic experience in obsessive-compulsive disorders: a pilot study. Verhaltenstherapie.

[18] Gershuny BS, Baer L, Jenike MA, Minichiello WE, Wilhelm S (2002). Comorbid posttraumatic stress disorder: impact on treatment outcome for obsessive-compulsive disorder. Am J Psychiatry.

[19] Gershuny BS, Baer L, Radomsky AS, Wilson KA, Jenike MA (2003). Connections among symptoms of obsessive-compulsive disorder and posttraumatic stress disorder: a case series. Behav Res Ther.

[20] Ghassemzadeh H, Shams G, Abedi J, Karamghadiri N, Ebrahimkhani N, Rajabloo M (2011). Psychometric properties of a Persian-language version of the Obsessive-Compulsive Inventory-Revised: OCI-R-Persian. Psychology.

[21] Grabe HJ, Ruhrmann S, Spitzer C, Josepeit J, Ettelt S, Buhtz F, Freyberger HJ (2008). Obsessive-compulsive disorder and posttraumatic stress disorder. Psychopathology.

[22] Hasanovic M, Srabovic S, Rasidovic M, Sehovic M, Hasanbasic E, Husanovic J, Hodzic R (2009). Psychosocial assistance to students with posttraumatic stress disorder in primary and secondary schools in post-war Bosnia Herzegovina. Psychiatr Danub.

[23] Helzer JE, Robins LN, McEvoy L (1987). Post-traumatic stress disorder in the general population. Findings of the epidemiologic catchment area survey. N Engl J Med.

[24] Hemmings SM, Lochner C, van der Merwe L, Cath DC, Seedat S, Stein DJ (2013). BDNF Val66Met modifies the risk of childhood trauma on obsessive-compulsive disorder. J Psychiatr Res.

[25] Huppert JD, Moser JS, Gershuny BS, Riggs DS, Spokas M, Filip J, Foa EB (2005). The relationship between obsessive-compulsive and posttraumatic stress symptoms in clinical and non-clinical samples. J Anxiety Disord.

[26] Kessler RC, Chiu WT, Demler O, Merikangas KR, Walters EE (2005). Prevalence, severity, and comorbidity of 12-month DSM-IV disorders in the National Comorbidity Survey Replication. Arch Gen Psychiatry.

[27] Lafleur DL, Petty C, Mancuso E, McCarthy K, Biederman J, Faro A, Geller DA (2011). Traumatic events and obsessive compulsive disorder in children and adolescents: is there a link?. J Anxiety Disord.

[28] Lorant V, Croux C, Weich S, Deliege D, Mackenbach J, Ansseau M (2007). Depression and socio-economic risk factors: 7-year longitudinal population study. Br J Psychiatry.

[29] Marshall GN, Schell TL, Elliott MN, Berthold SM, Chun CA (2005). Mental health of Cambodian refugees 2 decades after resettlement in the United States. JAMA.

[30] Mathews CA, Kaur N, Stein MB (2008). Childhood trauma and obsessive-compulsive symptoms. Depress Anxiety.

[31] Mathis MA, Alvarenga P, Funaro G, Torresan RC, Moraes I, Torres AR, Hounie AG (2011). Gender differences in obsessive-compulsive disorder: a literature review. Rev Bras Psiquiatr.

[32] Mollica RF, Caspi-Yavin Y, Bollini P, Truong T, Tor S, Lavelle J (1992). The Harvard Trauma Questionnaire. Validating a cross-cultural instrument for measuring torture, trauma, and posttraumatic stress disorder in Indochinese refugees. J Nerv Ment Dis.

[33] Mollica RF, Wyshak G, de Marneffe D, Khuon F, Lavelle J (1987). Indochinese versions of the Hopkins symptom checklist-25: a screening instrument for the psychiatric care of refugees. Am J Psychiatry.

[34] Mollica RF, Wyshak G, Marneffe DD, Tu B, Yang T, Khuon F (1991). Hopkins symptom checklist 25.

[35] Morina N, Reschke K, Hofmann SG (2011). Long-term outcomes of war-related death of family members in Kosovar civilian war survivors. Death Stud.

[36] Morina N, Rushiti F, Salihu M, Ford JD (2010). Psychopathology and well-being in civilian survivors of war seeking treatment: a follow-up study. Clin Psychol Psychother.

[37] Morina N, von Lersner U, Prigerson HG (2011). War and bereavement: consequences for mental and physical distress. PLoS One.

[38] Nacasch N, Fostick L, Zohar J (2011). High prevalence of obsessive-compulsive disorder among posttraumatic stress disorder patients. Eur Neuropsychopharmacol.

[39] Nelson BD, Fernandez WG, Galea S, Sisco S, Dierberg K, Gorgieva GS, Vlahov D (2004). War-related psychological sequelae among emergency department patients in the former Republic of Yugoslavia. BMC Med.

[40] Nijdam MJ, van der Pol MM, Dekens RE, Olff M, Denys D. Treatment of sexual trauma dissolves contamination fear: case report. Eur J Psychotraumatol*.* 2013; 4. doi: 10.3402/ejpt.v4i0.19157 10.3402/ejpt.v4i0.19157 PMC3540209 23304430

[41] Norris FH, Slone LB, Friedman MJ, Keane TM, Resick PA (2007). The epidemiology of trauma and PTSD. Handbook of PTSD: science and practice.

[42] Pitman RK (1993). Posttraumatic obsessive-compulsive disorder: a case study. Compr Psychiatry.

[43] Priebe S, Bogic M, Ajdukovic D, Franciskovic T, Galeazzi GM, Kucukalic A, Schutzwohl M (2010). Mental disorders following war in the Balkans: a study in 5 countries. Arch Gen Psychiatry.

[44] Priebe S, Matanov A, Jankovic Gavrilovic J, McCrone P, Ljubotina D, Knezevic G, Schutzwohl M (2009). Consequences of untreated posttraumatic stress disorder following war in former Yugoslavia: morbidity, subjective quality of life, and care costs. Croat Med J.

[45] Prorokovic A, Cavka M, Cubela Adoric V (2005). Psychosomatic and depressive symptoms in civilians, refugees, and soldiers: 1993–2004 longitudinal study in Croatia. Croat Med J.

[46] Riggs DS (2000). Treatment of concurrent PTSD and OCD: a commentary on the case of Howard. Cogn Behav Pract.

[47] Sasson Y, Dekel S, Nacasch N, Chopra M, Zinger Y, Amital D, Zohar J (2005). Posttraumatic obsessive-compulsive disorder: a case series. Psychiatry Res.

[48] Schick M, Morina N, Klaghofer R, Schnyder U, Mueller J. Trauma, mental health and intergenerational associations in Kosovar Families 11 years after the war. Eur J Psychotraumatol. 2013; 4. doi: 10.3402/ejpt.v4i0.21060 10.3402/ejpt.v4i0.21060 PMC3744842 23956820

[49] Schubert CC, Punamäki R-L (2011). Mental health among torture survivors: cultural background, refugee status and gender. Nord J Psychiatry.

[50] Speckens AE, Hackmann A, Ehlers A, Cuthbert B (2007). Imagery special issue: intrusive images and memories of earlier adverse events in patients with obsessive compulsive disorder. J Behav Ther Exp Psychiatry.

[51] Steel Z, Chey T, Silove D, Marnane C, Bryant RA, van Ommeren M (2009). Association of torture and other potentially traumatic events with mental health outcomes among populations exposed to mass conflict and displacement. JAMA.

[52] Ventevogel P, De Vries G, Scholte WF, Shinwari NR, Faiz H, Nassery R, Olff M (2007). Properties of the Hopkins Symptom Checklist-25 (HSCL-25) and the Self-Reporting Questionnaire (SRQ-20) as screening instruments used in primary care in Afghanistan. Soc Psychiatry Psychiatr Epidemiol.

[53] Wittchen HU, Jacobi F, Rehm J, Gustavsson A, Svensson M, Jonsson B, Steinhausen HC (2011). The size and burden of mental disorders and other disorders of the brain in Europe 2010. Eur Neuropsychopharmacol.

[54] Woo CW, Kwon SM, Lim YJ, Shin MS (2010). The Obsessive-Compulsive Inventory-Revised (OCI-R): psychometric properties of the Korean version and the order, gender, and cultural effects. J Behav Ther Exp Psychiatry.

[55] Xue C, Ge Y, Tang B, Liu Y, Kang P, Wang M, Zhang L (2015). A meta-analysis of risk factors for combat-related PTSD among military personnel and veterans. PLoS One.

